# HLA-A*0206 with TLR3 Polymorphisms Exerts More than Additive Effects in Stevens-Johnson Syndrome with Severe Ocular Surface Complications

**DOI:** 10.1371/journal.pone.0043650

**Published:** 2012-08-17

**Authors:** Mayumi Ueta, Katsushi Tokunaga, Chie Sotozono, Hiromi Sawai, Gen Tamiya, Tsutomu Inatomi, Shigeru Kinoshita

**Affiliations:** 1 Department of Ophthalmology, Kyoto Prefectural University of Medicine, Kyoto, Japan; 2 Research Center for Inflammation and Regenerative Medicine, Faculty of Life and Medical Sciences, Doshisha University, Kyoto, Japan; 3 Department of Human Genetics, Graduate School of Medicine, University of Tokyo, Tokyo, Japan; 4 Advanced Molecular Epidemiology Research Institute, Faculty of Medcine, Yamagata University, Yamagata, Japan; National Institute of Infectious Diseases, Japan

## Abstract

**Background:**

Stevens-Johnson syndrome (SJS) is an acute inflammatory vesiculobullous reaction of the skin and mucosa, often including the ocular surface, and toxic epidermal necrolysis (TEN) occurs with its progression. Although SJS/TEN is thought to be initiated by certain types of medication coupled with possible infection. In the present study we examined the multiplicative interaction(s) between HLA-A*0206 and 7 Toll-like receptor 3 (TLR3) Single-nucleotide polymorphisms (SNPs) in patients with SJS/TEN.

**Principal Findings:**

We analyzed the genotypes for HLA-A and 7 TLR3 SNPs in 110 Japanese SJS/TEN patients with severe ocular complications and 206 healthy volunteers to examine the interactions between the two loci. We found that HLA-A*0206 exhibited a high odds ratio for SJS/TEN (carrier frequency: OR = 5.1; gene frequency: OR = 4.0) and that there was a strong association with TLR3 rs.5743312T/T SNP (OR = 7.4), TLR3 rs.3775296T/T SNP (OR = 5.8), TLR3 rs.6822014G/G SNP (OR = 4.8), TLR3 rs.3775290A/A SNP (OR = 2.9), TLR3 rs.7668666A/A SNP (OR = 2.7), TLR3 rs.4861699G/G SNP (OR = 2.3), and TLR3 rs.11732384G/G SNP (OR = 1.9). There was strong linkage disequilibrium (LD) between rs.3775296 and rs.5743312 and between rs.7668666 and rs.3775290. The results of interaction analysis showed that the pair, HLA-A*0206 and TLR3 SNP rs3775296T/T, which exhibited strong LD with TLR3 rs.5743312, exerted more than additive effects (OR = 47.7). The other pairs, HLA-A*0206 and TLR3 rs.3775290A/A SNP (OR = 11.4) which was in strong LD with TLR3 rs7668666A/A SNP, and TLR3 rs4861699G/G SNP (OR = 7.6) revealed additive effects. Moreover, the combination HLA-A*0206 and TLR3 rs3775296T/T was stronger than the TLR3 rs6822014G/G and TLR3 rs3775290A/A pair, which reflected the interactions within the TLR3 gene alone.

**Significance:**

By interaction analysis, HLA-A*0206 and TLR3 SNP rs3775296T/T, which were in strong LD with TLR3 SNP rs5743312T/T, manifested more than additive effects that were stronger than the interactions within the TLR3 gene alone. Therefore, multiplicative interactions of HLA-A and TLR3 gene might be required for the onset of SJS/TEN with ocular complications.

## Introduction

Stevens-Johnson syndrome (SJS) is an acute inflammatory vesiculobullous reaction of the skin and mucous membranes. It was first described in 1922 by Stevens and Johnson, [1] both pediatricians, who encountered 2 boys aged 8 and 7 who manifested an extraordinary, generalized skin eruption, persistent fever, inflamed buccal mucosa, and severe purulent conjunctivitis resulting in marked visual disturbance. Subsequently, other pediatricians reported that SJS was associated with infectious agents such as *Mycoplasma pneumoniae*, [2] and a viral etiology involving herpes simplex virus, Epstein-Barr virus, cytomegalovirus, and varicella zoster virus [3]. On the other hand, dermatologists claimed that more than 100 different drugs were implicated in eliciting SJS and its severe form, toxic epidermal necrolysis (TEN) [4], [5]. The annual incidence of SJS and TEN has been estimated to be 0.4–1 and 1–6 cases per million persons, respectively; [6], [7] the reported mortality rate is 3% and 27%, respectively [8]. Although rare, these reactions have high morbidity and mortality rates, and often result in severe and definitive sequelae such as vision loss. SJS/TEN is one of the most devastating ocular surface diseases leading to corneal damage and loss of vision. The reported incidence of ocular complications in SJS/TEN is 50–68% [7], [8].

In the acute stage, patients manifest vesiculobullous lesions of the skin and mucosa, especially that of the eyes and mouth, and severe conjunctivitis. The loss of finger nails in the acute or subacute stage due to paronychia was observed, has been observed in almost all SJS/TEN patients with severe ocular surface complications [9], [10], [11], [12].

In the chronic stage, despite healing of the skin lesions, ocular surface complications such as conjunctival invasion into the cornea [10], [11], [12], [13], [14], [15], [16], [17], [18]. It is also reported that lid margin keratinization and tarsal scarring, together with lipid tear deficiency, contributes to corneal complications because of blink-related microtrauma [19].

Elsewhere we reported that the frequency of carriers of the HLA-A*0206 antigen is significantly higher among Japanese patients with severe ocular surface complications than in other populations [18], [20]. Our single nucleotide polymorphism (SNP) association analysis of candidate genes documented the associated polymorphisms of several immune-related genes including *TLR3*, [12], [17] *IL4R*, [14], [16] *IL13*, [16] and *FasL* [15]in Japanese SJS/TEN patients with severe ocular surface complications. To elucidate the detailed pathophysiology of SJS/TEN we performed a genome-wide association study of SJS/TEN patients and found associations between 6 SNPs in the prostaglandin E receptor 3 (EP3) gene (*PTGER3*) and SJS/TEN accompanied by severe ocular surface complications [11]. Moreover, gene-gene interaction analysis in SJS/TEN patients with severe ocular surface complications revealed that the interaction between *TLR3* and *PTGER3* exerted SJS/TEN susceptibility effects, and there was a functional interaction between TLR3 and EP3 in a murine experimental allergic conjunctivitis model. [12].

**Table 1:** Association between HLA-A*0206 and SJS/TEN with ocular complications.

HLA-A	Carrier frequency				Gene frequency			
	SJS(n = 110)	Normal(n = 206)	p-value(χ^2^)	OddsRatio	SJS(n = 220)	Normal(n = 412)	p-value(χ^2^)	OddsRatio
*0206	46.4% (51/110)	14.6% (30/206)	6.9×10^−10^	5.07	24.1% (53/220)	7.3% (30/412)	2.5×10^−9^	4.04
*0101	0% (0/110)	1.4% (3/206)	0.2	–	0% (0/220)	0.7% (3/412)	0.2	–
*0201	26.4% (29/110)	21.4% (44/206)	0.3	–	14.5% (32/220)	11.4% (47/412)	0.3	–
*0207	9.1% (10/110)	7.8% (16/206)	0.7	–	4.5% (10/220)	3.9% (16/412)	0.7	–
*0210	0% (0/110)	1.0% (2/206)	0.3	–	0% (0/220)	0.5% (2/412)	0.3	–
*0301	2.7% (3/110)	1.4% (3/206)	0.4	–	1.4% (3/220)	0.7% (3/412)	0.4	–
*0302	0% (0/110)	0.5% (1/206)	0.5	–	0% (0/220)	0.2% (1/412)	0.5	–
*1101	7.3% (8/110)	18.4% (38/206)	7.3×10^−3^	0.35	3.6% (8/220)	9.2% (38/412)	1.0×10^−2^	0.37
*1102	0% (0/110)	0.5% (1/206)	0.5	–	0% (0/220)	0.2% (1/412)	0.5	–
*2402	45.5% (50/110)	60.7% (125/206)	9.5×10^−3^	0.54	25.0% (55/220)	36.7% (151/412)	2.9×10^−3^	0.58
*2420	0% (0/110)	0.5% (1/206)	0.5	–	0% (0/220)	0.2% (1/412)	0.5	–
*2601	9.1% (10/110)	12.6% (26/206)	0.3	–	4.5% (10/220)	6.6% (27/412)	0.3	–
*2602	5.5% (6/110)	2.9% (6/206)	0.3	–	2.7% (6/220)	1.7% (7/412)	0.4	–
*2603	1.8% (2/110)	7.8% (16/206)	3.0×10^−2^	0.2	0.9% (2/220)	3.9% (16/412)	3.2×10^−2^	0.2
*2605	0% (0/110)	0.5% (1/206)	0.5	–	0% (0/220)	0.2% (1/412)	0.5	–
*2901	0% (0/110)	1.9% (4/206)	0.1	–	0% (0/220)	1.0% (4/412)	0.1	–
*3001	0.9% (1/110)	0% (0/206)	0.2	–	0.5% (1/220)	0% (0/412)	0.2	–
*3101	13.6% (15/110)	16.5% (34/206)	0.5	–	6.8% (15/220)	8.3% (34/412)	0.5	–
*3201	0% (0/110)	0.5% (1/206)	0.5	–	0% (0/220)	0.2% (1/412)	0.5	–
*3303	22.7% (25/110)	14.1% (29/206)	0.05	–	11.4% (25/220)	7.0% (29/412)	0.06	–

In the present study we examined the multiplicative interaction(s) between HLA-A*0206 and 7 TLR3 SNPs (rs3775296 (uSNP), rs5743312 (iSNP), rs6822014 (gSNP), rs3775290 (sSNP), rs7668666 (iSNP), rs11732384 (iSNP), and rs4861699 (gSNP)) associated with the SJS/TEN patients [12], [17] as the onset of SJS/TEN was associated not only with the administration of drugs but also with putative viral syndromes [10], [11], [12], [17]. HLA-A is a component of HLA class I, which resides on the surface of all nucleated cells and alerts the immune system that the cell may be infected by a virus, thereby targeting the cell for destruction. TLR3 recognises viral double-stranded RNA [21].

## Results

We analyzed the genotypes for HLA-A and 7 TLR3 SNPs in 110 Japanese SJS/TEN patients with severe ocular complications and 206 healthy volunteers to examine the interactions between the two loci.

**Table 2:** Association between TLR3 SNPs and SJS/TEN with ocular complications.

rs number of SNP	Genotypes		Case (N = 110)	Control (N = 206)	Allele 1 vs. Allele 2	Genotype 11 vs. 12+22	Genotype 11+12 vs. 22
					P-value^a^	P-value^a^	P-value^a^
					OR^b^	OR^b^	OR^b^
					(95%CI^c^)	(95%CI^c^)	(95%CI^c^)
rs4861699	11	G/G	65/110 (59.1%)	79/206 (38.3%)	0.0016	4.2×10^−4^	0.28
	12	G/A	36/110 (32.7%)	102/206 (49.5%)	1.80	2.32	1.55
	22	A/A	9/110 (8.2%)	25/206 (12.1%)	(1.25–2.59)	(1.45–3.72)	(0.70–3.45)
rs6822014	11	A/A	55/110 (50.0%)	127/206 (61.7%)	8.9×10^−4^	0.046	1.2×10^−4^
	12	A/G	37/110 (33.6%)	71/206 (34.5%)	0.54	0.62	0.21
	22	G/G	18/110 (16.4%)	8/206 (3.9%)	(0.37–0.78)	(0.39–0.99)	(0.09–0.49)
rs11732384	11	G/G	72/110 (65.5%)	103/206 (50.0%)	0.029	0.0085	0.88
	12	G/A	31/110 (28.2%)	89/206 (43.2%)	1.54	1.89	1.07
	22	A/A	7/110 (6.4%)	14/206 (6.8%)	(1.04–2.28)	(1.17–3.06)	(0.42–2.74)
rs3775296	11	G/G	49/110 (44.5%)	109/206 (52.9%)	0.0020	0.16	8.2×10^−6^
	12	G/T	40/110 (36.4%)	89/206 (43.2%)	0.58	0.71	0.17
	22	T/T	21/110 (19.1%)	8/206 (3.9%)	(0.40–0.82)	(0.45–1.14)	(0.07–0.40)
rs5743312	11	C/C	52/110 (47.3%)	115/206 (55.8%)	0.0014	0.15	2.5×10^−6^
	12	C/T	38/110 (34.5%)	85/206 (41.3%)	0.56	0.71	0.14
	22	T/T	20/110 (18.2%)	6/206 (2.9%)	(0.39–0.80)	(0.45–1.13)	(0.05–0.35)
rs7668666	11	C/C	36/110 (32.7%)	83/206 (40.3%)	0.0085	0.19	0.0012
	12	C/A	47/110 (42.7%)	101/206 (49.0%)	0.64	0.72	0.37
	22	A/A	27/110 (24.5%)	22/206 (10.7%)	(0.46–0.89)	(0.44–1.17)	(0.20–0.68)
rs3775290	11	G/G	38/110 (34.5%)	82/206 (39.8%)	0.016	0.36	7.1×10^−4^
	12	G/A	45/110 (40.9%)	103/206 (50.0%)	0.66	0.80	0.35
	22	A/A	27/110 (24.5%)	21/206 (10.2%)	(0.48–0.93)	(0.50–1.29)	(0.18–0.65)

a *P*-value for allele or genotype frequency comparisons between cases and controls using the chi-square test.

b OR, odds ratio.

c CI, confidence interval.

**Figure 1:**
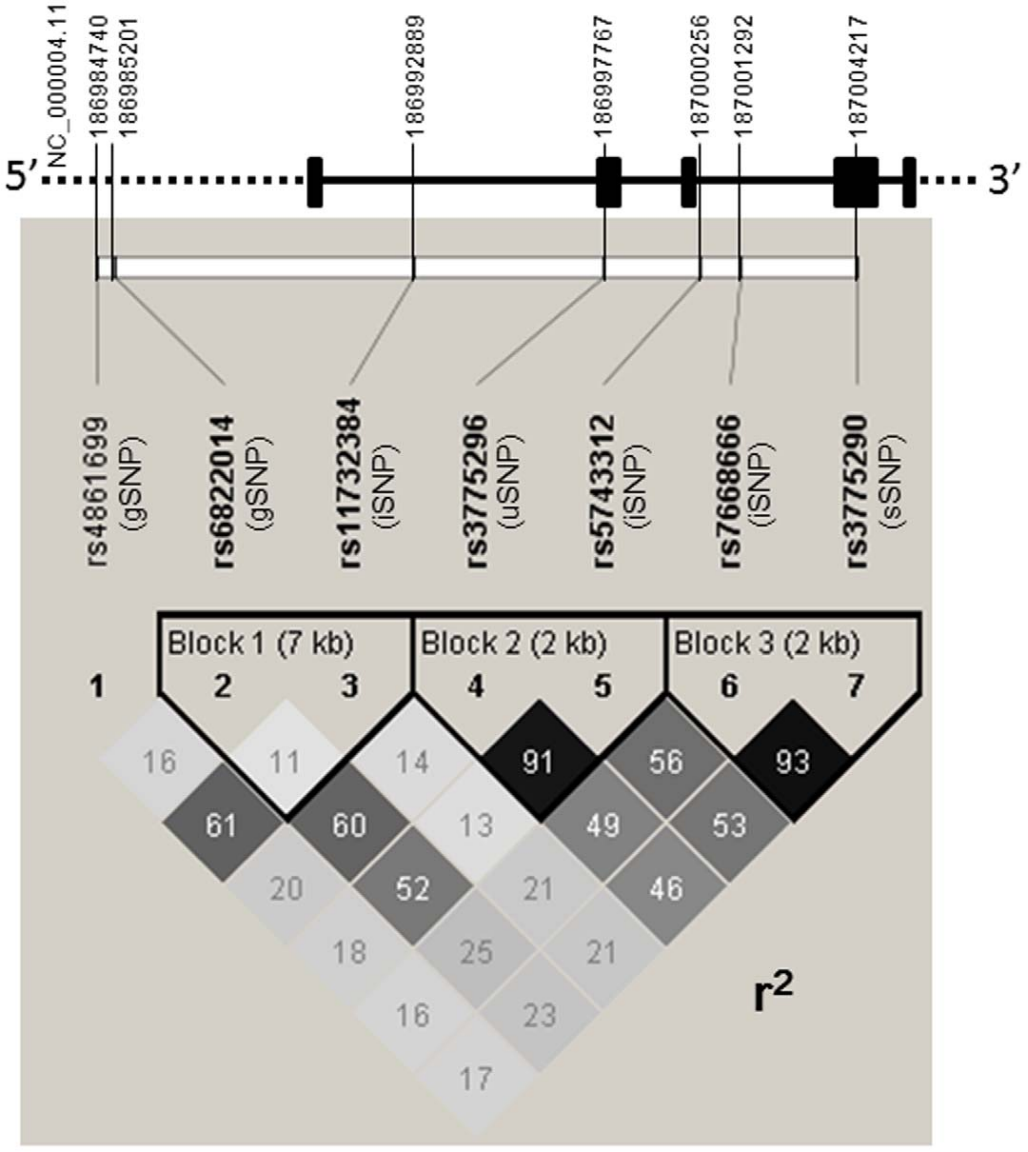
Linkage disequilibria among the 7 *TLR3* SNPs. Strong linkage disequilibrium was observed between rs.3775296 and rs.5743312, and between rs.7668666 and rs.3775290.

We found that HLA-A*0206 exhibited a high odds ratio for SJS/TEN (carrier frequency: p = 6.9×10^−10^, OR = 5.1; gene frequency: p = 2.5×10^−9^, OR = 4.0) (Table 1).

We also found that there was a strong association with TLR3 rs.5743312T/T SNP (T/T vs T/C+C/C: p = 2.5×10^−6^, OR = 7.4), TLR3 rs.3775296T/T SNP (T/T vs T/G+G/G: p = 8.2×10^−6^, OR = 5.8), TLR3 rs.6822014G/G SNP (G/G vs G/A+A/A: p = 1.2×10^−4^, OR = 4.8), TLR3 rs.3775290A/A SNP (A/A vs A/G+G/G: p = 7.1×10^−4^, OR = 2.9), TLR3 rs.7668666A/A SNP (A/A vs A/G+G/G: p = 1.2×10^−3^, OR = 2.7), TLR3 rs.4861699G/G SNP (G/G vs G/A+A/A: p = 4.2×10^−4^, OR = 2.3), and TLR3 rs.11732384G/G SNP (G/G vs G/A+A/A: p = 8.5×10^−3^, OR = 1.9) (Table 2). All SNPs were in Hardy-Weinberg equilibrium (p>0.01) in the samples from patients and the controls. Based on the squared correlation coefficient r^2^, we investigated the linkage disequilibrium (LD) among the *TLR3* SNPs. We found strong LD between rs.3775296 and rs.5743312 (D’ = 1, r^2^ = 0.911), and between rs.7668666 and rs.3775290 (D’ = 0.973, r^2^ = 0.934) (Fig. 1).

**Table 3:** Interaction analysis between HLA-A*0206 and various TLR3 SNPs.

HLA-A*0206	TLR3 SNP	SJS patients (N = 110)	Controls (N = 206)	OR	p-value		Standardized OR
HLA-A*0206 & TLR3 rs3775296 T/T							
+	+	11/110 (10%)	0/206 (0%)	47.7*	6.5×10^−6^ **		262.7
+	−	40/110 (36.4%)	30/206 (14.6%)	3.4	8.8×10^−6^		18.5
−	+	10/110 (9.1%)	8/206 (3.9%)	2.5	0.057		13.6
−	−	49/110 (44.5%)	168/206 (81.6%)	0.18	1.4×10^−11^		1
HLA-A*0206 & TLR3 rs6822014G/G							
+	+	8/110 (7.3%)	3/206 (1.5%)	5.3**	0.019**		32.3
+	−	43/110 (39.1%)	27/206 (13.1%)	4.3	1.2×10^−7^		25.9
−	+	10/110 (9.1%)	5/206 (2.4%)	4.0**	0.012**		24.5
−	−	49/110 (44.5%)	171/206 (83.0%)	0.16	1.4×10^−12^		1
HLA A*0206 & TLR3 rs3775290A/A							
+	+	16/110 (14.5%)	3/206 (1.5%)	11.4**		7.4×10^−6^ **	49.0
+	−	35/110 (31.8%)	27/206 (13.1%)	3.1		6.6×10^−5^	13.2
−	+	11/110 (10%)	18/206 (8.7%)	1.2		0.71	4.9
−	−	48/110 (43.6%)	158/206 (76.7%)	0.24		4.2×10^−9^	1
HLA A*0206 & TLR3 rs11732384G/G							
+	+	37/110 (33.6%)	16/206 (7.8%)	6.0	4.5×10^−9^		16.4
+	−	14/110 (12.7%)	14/206 (6.8%)	2	0.077		5.5
−	+	35/110 (31.8%)	87/206 (42.2%)	0.64	0.070		1.7
−	−	24/110 (21.8%)	89/206 (43.2%)	0.37	1.5×10^−4^		1
HLA A*0206 & TLR3 rs4861699 G/G							
+	+	33/110 (30%)	11/206 (5.3%)	7.6	1.6×10^−9^		25.7
+	−	18/110 (16.4%)	19/206 (9.2%)	1.9	0.060		6.5
−	+	32/110 (29.1%)	68/206 (33.0%)	0.83	0.48		2.8
−	−	27/110 (24.5%)	108/206 (52.4%)	0.30	1.8×10^−6^		1

* Woolf’s correction,

** Fisher’s exact test.

**Table 4:** Interaction analysis of two SNPs of the TLR3 SNPs (SJS > control and SJS >5).

Combination of 2 TLR3 SNPs		SJS (N = 110)	Controls (N = 206)	OR	p-value
rs3775296 T/T +	rs3775290 A/A +	19/110 (17.3%)	6/206 (2.9%)	7.0	6.6×10^−6^
rs11732384 G/G +	rs3775290 A/A +	27/110 (24.5%)	21/206 (10.2%)	2.9	7.1×10^−4^
rs6822014 G/G +	rs3775290 A/A +	15/110 (13.6%)	2/206 (1.0%)	16.1	2.0×10^−6^
rs4861699 G/G +	rs3775290 A/A +	26/110 (23.6%)	16/206 (7.8%)	3.7	7.5×10^−5^
rs11732384 G/G +	rs3775296 T/T +	21/110 (19.1%)	8/206 (3.9%)	5.8	8.2×10^−6^
rs6822014 G/G +	rs3775296 T/T +	17/110 (15.5%)	4/206 (1.9%)	9.2	4.3×10^−6^
rs4861699 G/G +	rs3775296 T/T +	21/110 (19.1%)	8/206 (3.9%)	5.8	8.2×10^−6^
rs6822014 G/G +	rs11732384 G/G +	18/110 (16.4%)	8/206 (3.9%)	4.8	1.2×10^−4^
rs4861699 G/G +	rs6822014 G/G +	18/110 (16.4%)	8/206 (3.9%)	4.8	1.2×10^−4^

Results of interaction analysis showed that the pair, HLA-A*0206 and TLR3 SNP rs3775296T/T, which exhibited strong LD with TLR3 rs.5743312, exerted more than additive effects. We found that while 11 of the 110 patients (10%) manifested both HLA-A*0206 and TLR3 rs3775296T/T SNP, none of the 206 controls did (p = 6.5×10^−6^, OR = 47.7, Woolf’s correction). The other pairs, HLA-A*0206 and TLR3 rs.3775290A/A SNP, which was in strong LD with TLR3 rs.7668666, or TLR3 rs4861699G/G SNP revealed additive effects: 16 of the 110 patients (14.5%) but only 3 of the 206 controls (1.5%) had both HLA-A*0206 and TLR3 rs.3775290A/A SNP (p = 7.4×10^−6^, OR = 11.4). In addition, 33 of the 110 patients (30%), compared to 11 of the 206 controls (5.3%), had both HLA-A*0206 and TLR3 rs.4861699G/G SNP (p = 1.6×10^−9^, OR = 7.6) (Table 3).

Moreover, to examine the interactions within the TLR3 gene alone we analyzed interactions between 2 each of 5 TLR3 SNPs (rs3775296, rs6822014, rs3775290, rs11732384, rs4861699). Combinations of high risk genotypes, on which the observed numbers in cases were greater than of the controls and greater than five, were analyzed. One of the 9 combinations, TLR3 rs6822014G/G and TLR3 rs3775290A/A, exerted more than additive effects (OR 16.1, p = 2.0×10^−6^) (Table 4). However, the combination HLA-A*0206 and TLR3 rs3775296T/T produced a stronger additive effect than it. In addition, we performed haplotype association analysis with the 7 TLR3 SNPs (rs4861699, rs6822014, rs11732384, rs3775296, rs5743312, rs7668666, rs3775290) and the 5 TLR3 SNPs (rs4861699, rs6822014, rs11732384, rs3775296, rs3775290), and found that no haplotype showed strong association (p<0.001) (Table S1). Thus, the haplotype associations appear to contribute little to the observed interactions.

## Discussion

To our knowledge, ours is the first report documenting the additive effects of HLA-A*0206 and TLR3 polymorphisms. Our interaction analysis showed that the pair HLA-A*0206 and TLR3 SNP rs3775296T/T, which was in strong LD with TLR3 rs.5743312, exerted more than additive effects, and that other pairs, HLA-A*0206 and TLR3 rs.3775290A/A SNP in strong LD with TLR3 rs.7668666, and TLR3 rs4861699G/G SNP exerted additive effects. Moreover, the combination HLA-A*0206 and TLR3 rs3775296T/T was stronger than the combination with TLR3 rs6822014G/G or TLR3 rs3775290A/A, the interactions within the TLR3 gene alone.

HLA-A, a component of HLA class I, alerts the immune system that the cell may be infected with a virus; TLR3 recognizes viral double-stranded RNA [21]. It is worth noting that about 80% of our SJS patients developed SJS after receiving treatment for the common cold with antibiotics, cold remedies, and/or NSAIDs; only about 5% of our SJS patient progressed to SJS after drug treatment to prevent the occurrence of convulsions [11], [12]. Moreover, our review of medical records revealed that 9 of the 11 patients with both HLA-A*0206 and TLR3 SNP rs3775296T/T (and rs.5743312T/T) developed SJS after receiving cold medicine, leading us to suspect that they already had a viral infection before taking the cold medicine. Particulars on the other 2 patients are unknown because they developed SJS during childhood.

Although the TLR3 SNPs exerting additive- or more than additive effects with HLA-A*0206 were u-, i-, or gSNPs and without amino acid changes, it is possible that TLR3 SNPs and HLA-A*0206 were involved in the onset of SJS with severe ocular surface complications. Moreover, their interaction might influence the host immune response against viral infection with drug treatments.

Earlier reports indicated regional differences in HLA associations. Although in Japanese SJS patients we were unable to detect the HLA-Bw44 antigen, a subgroup of HLA-B12 [19], [23], it was significantly increased in Caucasian SJS patients with ocular involvement [22].

On the other hand, the HLA-A*0206 antigen, which is not found in Caucasians [18], [19] was significantly increased in our Japanese SJS patients with ocular complications. While there might be ethnic differences in the association of SJS/TEN with HLA,[18], [19] specific combinations of genes and certain environmental factors may be required for the manifestation of this rare phenotype. [10], [11], [12], [18], [19].

Elsewhere [12] we reported that the epistatic interaction between TLR3 and PTGER3 confers an increased risk for SJS with ocular complications. Since SJS/TEN is a rare condition that probably has a complex genetic background, it is reasonable to posit that multiplicative interactions of genes such as HLA-A & TLR3, and TLR3 & PTGER3, are required for the phenotypic manifestation.

In summary, we show that HLA-A*0206 with TLR3 polymorphisms exerts more than additive effects in SJS with severe ocular surface complications and we suggest that gene-gene interactions should be considered in addition to major single-locus effects.

## Materials and Methods

### Patients

This study was approved by the institutional review board of Kyoto Prefectural University of Medicine and the University of Tokyo, Graduate School of Medicine. All experimental procedures were conducted in accordance with the principles of the Helsinki Declaration. The purpose of the research and the experimental protocols were explained to all participants, and their prior written informed consent was obtained.

Diagnosis of SJS/TEN was based on a confirmed history of acute onset of high fever, serious mucocutaneous illness with skin eruptions, and involvement of at least 2 mucosal sites including the ocular surface [9], [11], [12], [17], [18].

To investigate the gene-gene interaction between HLA-A*0206 and TLR3, we enrolled 110 SJS/TEN patients in the chronic or subacute phase; all presented with symptoms of ocular surface complications. None of the patients were relatives. The controls were 206 healthy volunteers. All participants and volunteers were Japanese residing in Japan. The average age of the 110 patients and 206 controls was 43.6±18.0 (SD) and 35.4±11.1 (SD) years, respectively. The male:female ratios in the patient and control groups were 42∶68 and 82∶124, respectively. Some of the SJS/TEN patients and controls in this study were subjects in our earlier reports [12], [17], [18], [19].

### TLR3 SNPs Genotyping

Genomic DNA was isolated from human peripheral blood at SRL Inc. (Tokyo, Japan). Genotyping for 2 SNPs of TLR3 (rs3775290, 3775296) was performed by PCR-direct sequencing as reported previously [17]. For direct sequencing, PCR amplification was conducted with AmpliTaq Gold DNA Polymerase (Applied Biosystems) for 35 cycles at 94°C for 1 min, annealing at 60°C for 1 min, and 72°C for 1 min on a commercial PCR machine (GeneAmp; Perkin-Elmer Applied Biosystems). The PCR products were reacted with BigDye Terminator v3.1 (Applied Biosystems) and sequence reactions were resolved on an ABI PRISM 3100 Genetic Analyzer (Applied Biosystems).

Genotyping for 5 SNPs of TLR3 (rs4861699, rs6822014, rs11732384, rs5743312, rs7668666) as performed using DigiTag2 assay [12]. Multiplex PCR was performed in 10 µl of Multiplex PCR buffer containing 25 ng genomic DNA, 25 nM of each multiplex primer mix, 200 µM of each dNTP, 2.25 mM MgCl_2_, and 0.4 U KAPA2G Fast HotStart DNA polymerase (Kapa Biosystems). Cycling was performed at 95°C for 3 min, followed by 40 cycles of 95°C for 15 s and 68°C for 2 min. The primers and probes used in this study previously were reported [12], [17].

### HLA-A Genotyping

For HLA-A genotyping, we performed polymerase chain reaction amplification followed by hybridization with sequence-specific oligonucleotide probes (PCR-SSO) using commercial bead-based typing kits (WAK Flow, Wakunaga, Hiroshima, Japan), as described previously [18], [19].

### Statistical Analysis

Statistical significance of the association with each SNP was assessed using Chi-square test or Fisher’s exact test on two-by-two contingency tables. When the value obtained for the control was 0 the odds ratio was calculated using Woolf’s correction.

Haploview software (ver. 4.2) was used to infer the linkage disequilibrium structure of the 7 TLR3 SNPs and to perform a haplotype analysis of TLR3 gene.

## Supporting Information

Table S1 **Haplotype analysis of TLR3 gene.** Haplotype association analysis with the 7 TLR3 SNPs (rs4861699, rs6822014, rs11732384, rs3775296, rs5743312, rs7668666, rs3775290) and the 5 TLR3 SNPs (rs4861699, rs6822014, rs11732384, rs3775296, rs3775290)
(DOCX)
