# Supplementary material for: HLA-A*0206 with TLR3 Polymorphisms Exerts More than Additive Effects in Stevens-Johnson Syndrome with Severe Ocular Surface Complications
Source: PLoS One. 2012 Aug 17;7(8):e43650. doi: 10.1371/journal.pone.0043650 (PMC3422242; doi:10.1371/journal.pone.0043650)
Supplement: Table S1 — Haplotype analysis of TLR3 gene. Haplotype association analysis with the 7 TLR3 SNPs (rs4861699, rs6822014, rs11732384, rs3775296, rs5743312, rs7668666, rs3775290) and the 5 TLR3 SNPs (rs4861699, rs6822014, rs11732384, rs3775296, rs3775290) (DOCX) [file pone.0043650.s001.docx]

**Table S1**

***Haplotype analysis of TLR3 gene***

| Block | Haplotype Frequencies | Case Frequencies | Control Frequencies | P Value |
| --- | --- | --- | --- | --- |
| Haplotypes of 7 SNPs (rs4861699, rs6822014, rs11732384, rs3775296, rs5743312, rs7668666, rs3775290) | | | | |
| GAGGCCG | 0.267 | 0.257 | 0.273 | 0.6651 |
| AAAGCCG | 0.240 | 0.186 | 0.269 | 0.02 |
| GGGTTAA | 0.201 | 0.271 | 0.163 | 0.0013 |
| GAGGCAA | 0.076 | 0.087 | 0.070 | 0.4442 |
| GAGTTAA | 0.063 | 0.051 | 0.069 | 0.3753 |
| AAGGCCG | 0.041 | 0.028 | 0.049 | 0.2191 |
| AAGGCAA | 0.040 | 0.032 | 0.044 | 0.4335 |
| GGGGCCG | 0.022 | 0.015 | 0.026 | 0.3819 |
| GGGTCCG | 0.017 | 0.018 | 0.017 | 0.9113 |
| GAAGCCG | 0.012 | 0.019 | 0.008 | 0.2312 |
| Haplotypes of 5 SNPs (rs4861699, rs6822014, rs11732384, rs3775296, rs3775290) | | | | |
| GAGGG | 0.266 | 0.257 | 0.271 | 0.7065 |
| AAAGG | 0.243 | 0.186 | 0.273 | 0.0147 |
| GGGTA | 0.201 | 0.272 | 0.163 | 0.0011 |
| GAGGA | 0.079 | 0.094 | 0.072 | 0.3302 |
| GAGTA | 0.065 | 0.056 | 0.069 | 0.5145 |
| AAGGG | 0.042 | 0.032 | 0.048 | 0.3242 |
| AAGGA | 0.039 | 0.028 | 0.045 | 0.2948 |
| GGGGG | 0.020 | 0.012 | 0.025 | 0.2882 |
| GGGTG | 0.029 | 0.044 | 0.020 | 0.079 |
| GAAGG | 0.011 | 0.016 | 0.008 | 0.3469 |
